# Exposure of Wild Boar to *Mycobacterium tuberculosis* Complex in France since 2000 Is Consistent with the Distribution of Bovine Tuberculosis Outbreaks in Cattle

**DOI:** 10.1371/journal.pone.0077842

**Published:** 2013-10-22

**Authors:** Céline Richomme, Mariana Boadella, Aurélie Courcoul, Benoît Durand, Antoine Drapeau, Yannick Corde, Jean Hars, Ariane Payne, Alexandre Fediaevsky, María Laura Boschiroli

**Affiliations:** 1 Anses, Nancy laboratory for rabies and wildlife, Malzéville, France; 2 SaBio-IREC (CSIC-UCLM-JCCM), Ciudad-Real, Spain; 3 University Paris-Est, Anses, Laboratory for animal health, Epidemiology unit, Maisons-Alfort, France; 4 University Paris-Est, Anses, Laboratory for animal health, Bovine tuberculosis national reference laboratory, Maisons-Alfort, France; 5 National Hunting and Wildlife Agency (ONCFS), Research department, Gières, France; 6 Lyon University, UMR CNRS 5558, Villeurbanne, France; 7 Ministry of Food, Agriculture and Fisheries, Directorate General for Food (DGAl), Paris, France; University College Dublin, Ireland

## Abstract

The Eurasian wild boar (*Sus scrofa*) is increasingly considered as a relevant actor in the epidemiology of animal tuberculosis (TB). Therefore, monitoring TB in wild boar becomes a key tool for establishing comprehensive control schemes for this disease. To estimate the exposure of free living wild boar to Mycobacterium tuberculosis complex (MTC) in France, a bovine-purified protein derivative based ELISA was used to test 2,080 archived serum samples of hunter-harvested animals in 58 French “départements”. Two cut-off values were used for diagnostic interpretation: 0.2, recommended by the manufacturer (specificity: 96.43%; sensitivity: 72.6%), and 0.5 (specificity: 100%; sensitivity: 64%). During the same period, at the 0.2 cut-off, global true seroprevalence was 5.9% (IC_95%_: 4.3%-7.7%) and 76% of the sampled “départements” had seropositive wild boar, including seven cattle TB-free “départements. At the 0.5 cut-off, global true seroprevalence was 2.2% (IC_95%_: 1.5-3.2) and positive wild boar belonged to 21% of the “départements”. All but one of these positive “départements” had reported at least one cattle TB outbreak since 2000. A good consistence between seropositive wild boar and TB outbreaks in cattle was found, especially at the 0.5 cut-off value (the mean distance to the nearest cattle TB outbreak was 13km and 27km for seropositive and seronegative wild boar, respectively; P<0.05). The use of an ELISA to detect MTC antibodies in wild boar has permitted the description of the geographic distribution of MTC contact in wild boar in France. Our results suggest that the ELISA could be used as a first screening tool to conduct TB surveillance in wild boar at a population level. High-risk wild boar populations (e.g. overabundant) could be tested and if identified positive by ELISA they should be surveyed in detail by combining pathology and culture.

## Introduction

Bovine tuberculosis (TB) is a zoonotic disease caused by *Mycobacterium bovis* and closely related members of the Mycobacterium tuberculosis complex (MTC). Members of the MTC can infect a broad range of hosts, including wildlife. In particular for *M. bovis*, different wild species have been recognized as TB reservoirs in different areas of the world, becoming a source of infection for domestic livestock and thus, impeding the eradication in cattle [1]. The relevance of wildlife TB reservoirs increases as prevalence in livestock decreases since spill-back from wildlife hinders eradication in domestic species. Moreover, wild ungulates are in continuous expansion both geographically and numerically in Europe, driven by game management practices and changes in agricultural production [2,3]. This expansion increases the general concern regarding the control of diseases at the wildlife-livestock interface [4]. Under these circumstances, monitoring TB in wildlife becomes a key tool for establishing comprehensive eradication schemes [5].

**Figure 1:**
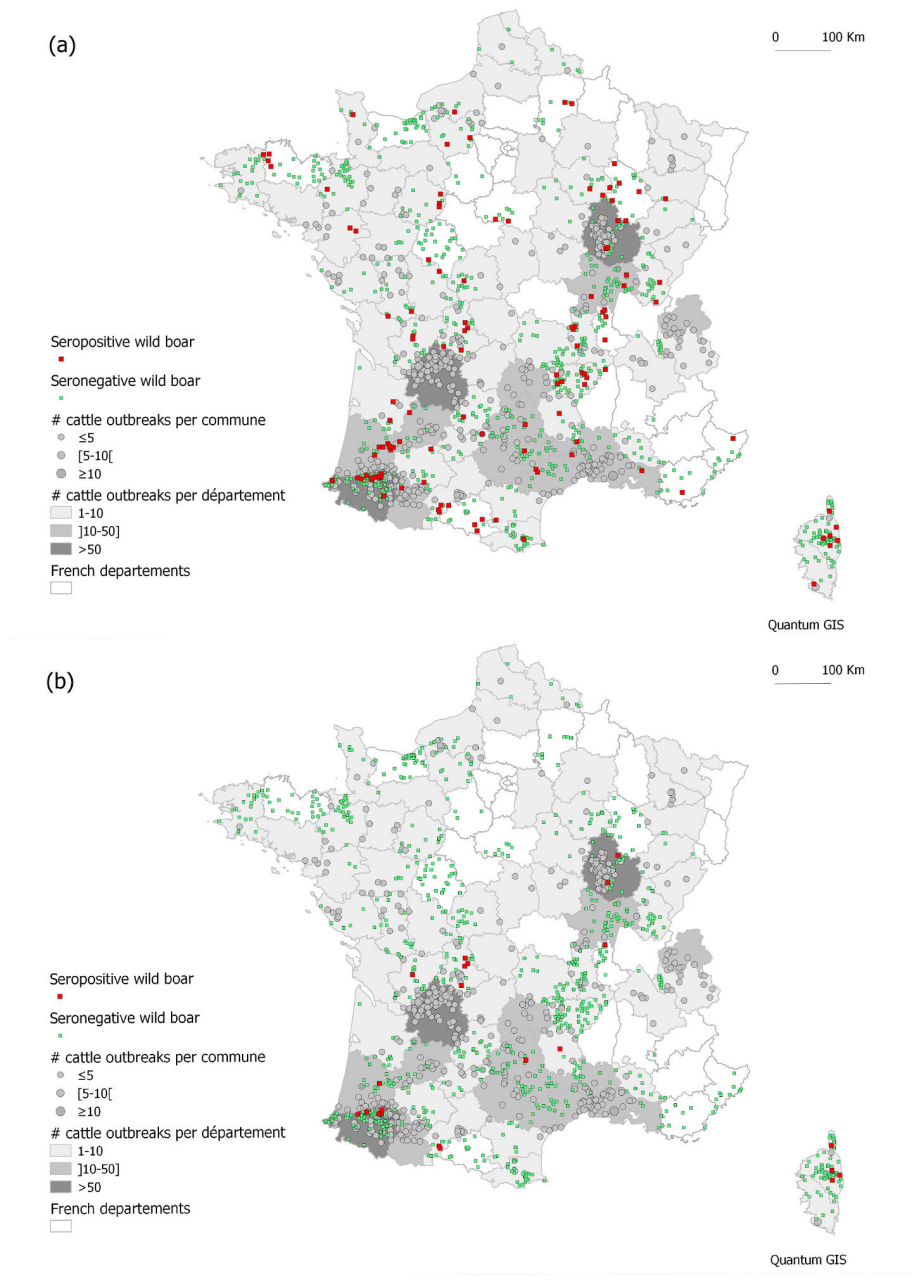
Distribution of the tested wild boar: green square= seronegative wild boar; red square = seropositive wild boar (a) when using the 0.2 cut-off and (b) when using the 0.5 cut-off. Wild boar location was affected to the centroid of sampling commune. Circles figure the cumulative number per commune of TB outbreaks in cattle between 2000 and 2010 (diameter proportional to the number of outbreaks during the period, from 1 to 13) and the colour in the “département” the cumulative intensity of TB outbreaks detected in cattle in the same period (white “département”: no outbreak between 2000 and 2010; intensity of grey proportional to the number of outbreaks, from 1 to 141).

In December 2000, the European Commission declared France as officially TB-free. Nevertheless, the number of infected herds has increased in a few French areas where several cattle outbreaks are still detected each year, especially in Côte d’Or, in the East, in Dordogne and in the Pyrénées-Atlantiques, in South-West (hard grey on Figure 1), or in Camargue, on the Mediterranean coast. The first cases of wildlife TB in France were detected in red deer (*Cervus elaphus*) and in Eurasian wild boar (*Sus scrofa*) in 2001 in the Brotonne-Mauny forest (Normandy, in the North-West) (Figure S1), a particular closed-environment area. In 2006, the TB prevalence in this small area reached 24% in red deer, which appeared to act as the primary host, and 42% in wild boar which appeared to act as a spillover host [6,7]. In 2003, a red deer was also found infected in Côte d’Or where grouped cases of wild boar and badgers (*Meles meles*) have been regularly identified since 2007 [8]. Elsewhere in France, sporadic TB cases in wild boar and/or badgers are detected in the vicinity of cattle outbreaks. The genotypes of *M. bovis* strains infecting both cattle and wildlife are identical [6,9], clearly indicating an epidemiological link between cattle, the environment and wildlife which could be involved in a common transmission cycle [10]. Whereas three potential wild MTC reservoirs are suspected - wild boar, red deer, and badger, their role in the French TB epidemiological scenario is still not clearly understood. 

In some ecological conditions, wild boar can be an important TB reservoir as demonstrated in the Mediterranean habitats of south-western Spain, where some favoring factors are present: habitat factors such as summer droughts that favor aggregation, but also artificial feeding and watering, and overabundant populations [11-13]. In such epidemiological situation prevalence in wildlife can be high[14] and it seems that wild boar could play a role of disseminator of TB. Actually, in the remaining parts of peninsular Spain, although wild boar management is less human-linked in, wild boar populations are also increasing [15], and the first wild boar TB cases have recently been reported in Asturias [16,17], suggesting a possible TB expansion due to this species northwards. Moreover, *Sus scrofa* is a suitable indicator of TB environmental contamination [18]. As the role of wildlife as a TB reservoir on the one hand and as a sentinel on the other hand is increasingly being recognized the development of new suitable tools for monitoring TB in wildlife has become crucial [5]. 

In order to test antibodies against members of the MTC in wild boar, an indirect enzyme linked immunosorbert assay (ELISA) using bovine-purified protein derivative (bPPD) has recently been developed [19,20]. This ELISA positively correlates with TB lesions in wild boar under experimental conditions [21] and therefore has become an inexpensive and observer-independent tool for MTC contact monitoring in this species [22], which is besides a robust test where even simply to harvest blood samples can be used. 

Bearing in mind the use of abovementioned ELISA to explore the epidemiology of MTC in wild boar at a large scale in France, the first aim of the present study was to retrospectively estimate the exposure of free living wild boar in France to MTC by using archived serum samples. The second aim was to determine if this exposure was consistent with another indicator of the presence of *M. bovis*, TB outbreaks in cattle, as a proxy to assess if the ELISA could be used as a tool to perform wide TB surveillance at a population level in wild boar.

## Material and Methods

### Ethics statement

All samples were provided by hunters who held the appropriate permits for hunting wild boar in season. All sampling was in complete agreement with national and European regulations. No ethical approval was deemed necessary. This study did not involve purposeful killing of animals.

### Wild boar samples

Sera from 2,080 hunter-harvested wild boar were analysed. They were collected for previous investigations concerning other diseases in 58 out of the 96 mainland French administrative units called “départments” and in the island of Corsica (Table S1). From them, 1,653 were from the Anses serobank (Nancy, France) and had been collected between 2000 and 2004 in 55 “départements” during the national serological investigation on *Brucella*, *Trichinella* and Aujeszky’s disease (ONCFS- hunting federations – DGAl - Anses program; 1 to 105 samples per “département”) [23]. Other 427 sera had been collected in 2009 and 2010, 285 in 5 continental “départements” for a trichinellosis survey (ONCFS- hunting federation – DGAl - Anses program; 21 to 108 samples per “département”) [24] and 142 in the 2 “départements” of Corsica for an hepatitis E survey (Anses and French national institute for agricultural research program; 13 and 129 samples). Only 4 “départements” presented samples collected in both periods (Aveyron, Ille-et-Vilaine, Pyrénées-Atlantiques, Haute-Corse; Figure S1) (n=451). All serum samples were stored at -20°C until the present study. 

The sampling commune (smallest administrative unit) was known for 2,008 individuals, and the gender for 2,006 animals (1,063 males and 943 females). Age classes of biological meaning were known for 2,008 animals: individuals less than 12 months were classified as juveniles (n=655) and older as adults (n=1363). 

### Serological test

Serum samples, that had previously gone through less than five freeze - thaw cycles [25], were tested by means of an indirect ELISA using bovine-purified protein derivative (bPPD) following the manufacturer’s instructions (TB-ELISA, Vacunek, Spain). Eleven naturally *M. bovis* infected, culture-confirmed, and previously bPPD-ELISA tested wild boar, were used as positive controls [20]. Following the manufacturer’s instructions, results were expressed as an ELISA index (EI) that was calculated using the following formula: 

Sample EI = mean sample OD (405nm-450nm)/mean positive control OD (405nm-450nm) 

Two cut-off values were used: 0.2, which is the cut-off recommended by the manufacturer (specificity: 96.43%; sensitivity: 72.6%), and 0.5, which was established using the Receiver-Operating Curve [19] to target a specificity of 100% (sensitivity: 65%).

### Data analysis

Data from the two periods (2000-2004 and 2009-2010) were merged after verification that seroprevalence did not significantly differ from a period to the other for the “départements” regardless of the prevalence of cattle TB, where samples had been collected during both periods. Apparent and true seroprevalences, which take into account the sensitivity and the specificity of the ELISA at the chosen cut-off values, were calculated. The Sterne’s exact method [26] was used to estimate 95% apparent and true prevalence confidence intervals (CI 95%). Exploratory analysis of host factors modulating TB exposure level (age class and sex) was carried out using Fischer’s test. 

We also assessed if exposure to MTC in wild boar was consistent with another indicator of the presence of MTC bacteria, i.e. TB cattle outbreaks due to *M. bovis* (n=803 for the period 2000-2010; Cattle TB data from the French Ministry of Agriculture and the bovine tuberculosis National reference laboratory). For each wild boar, we first calculated the Euclidian distance between the centroid from the commune of sampling (finest scale of spatial position available) to the centroid of the commune of the nearest cattle outbreak for the 2000–2010 period, hereafter called d-outbreak. 

The proximity of wild boar to cattle TB outbreaks was analyzed using a bootstrap method. The null hypothesis was that the serological status of animals was independent of their d-outbreak. The analysis was focused on the average d-outbreak in seropositive wild boar. The observed value of this statistic of interest was first computed. A resampling procedure was then used to simulate samples from the data, under the null hypothesis. A bootstrap sample was obtained by allocating a randomly generated permutation of the serological results to wild boar. One thousand bootstrap samples were generated, and, for each of them, the statistic of interest was computed. The corresponding distribution was finally examined to determine the empirical p-value of the null hypothesis test: this p-value was the proportion of the samples (simulated under the null hypothesis) for which the statistic of interest was below the actual value (computed from the data).

Statistical tests were considered significant if the p-value (p) was < 0.05. All statistical analyses were performed using R software [27], true prevalence calculation using the “epi.prev” function (epiR package).

## Results

The seropositivity in the 4 “départements” sampled in the two periods did not differ significantly from Period 1 to Period 2 (p > 0.05 for each “département” – Aveyron, Ille-et-Vilaine, Pyrénées-Atlantiques, Haute-Corse - with both cut-off values). 

**Figure 2:**
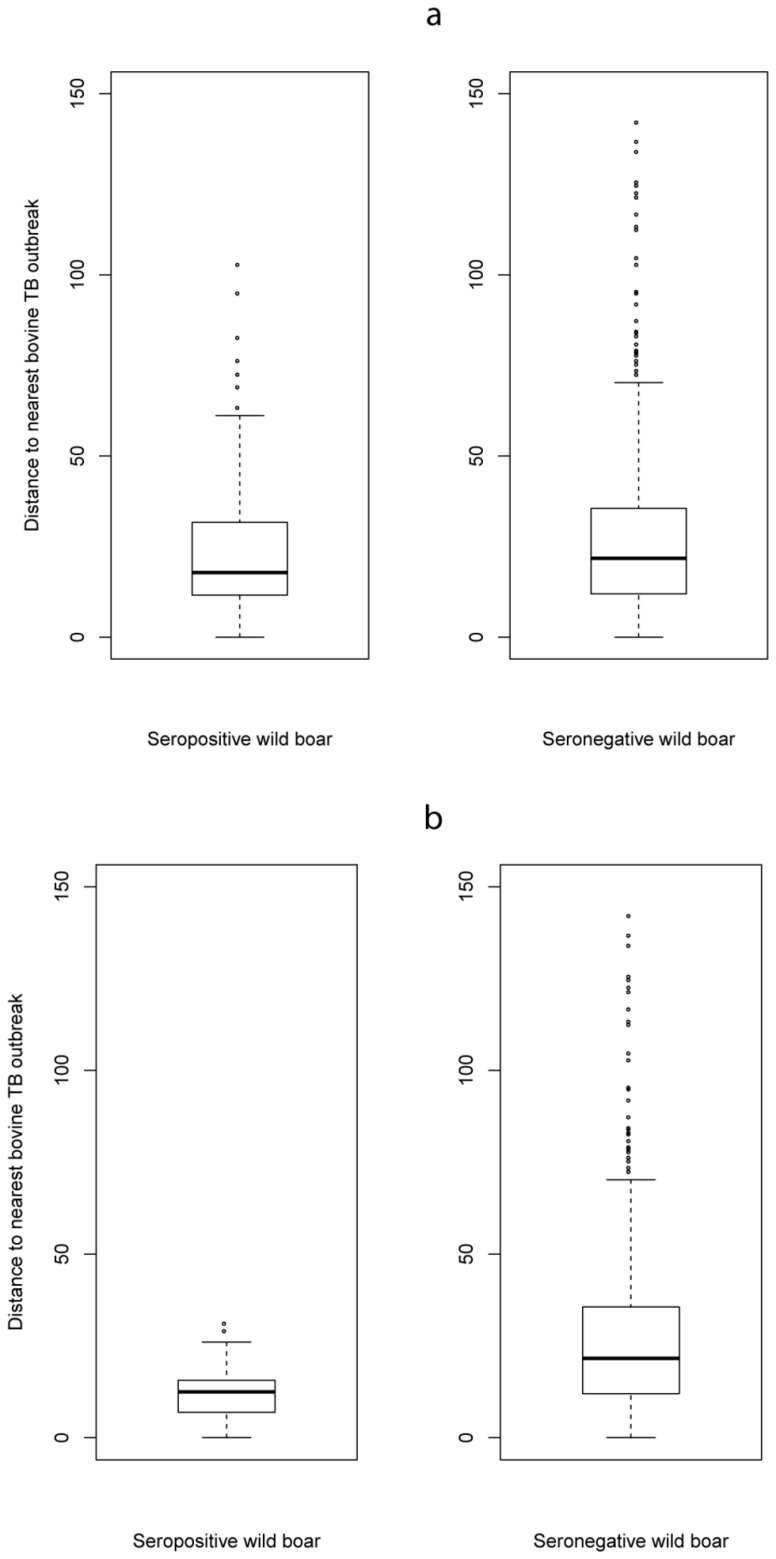
Distances between wild boar and the nearest TB outbreak in cattle: (a) bPPD ELISA cut-off 0.2, (b) bPPD ELISA cut-off 0.5.

**Figure 3:**
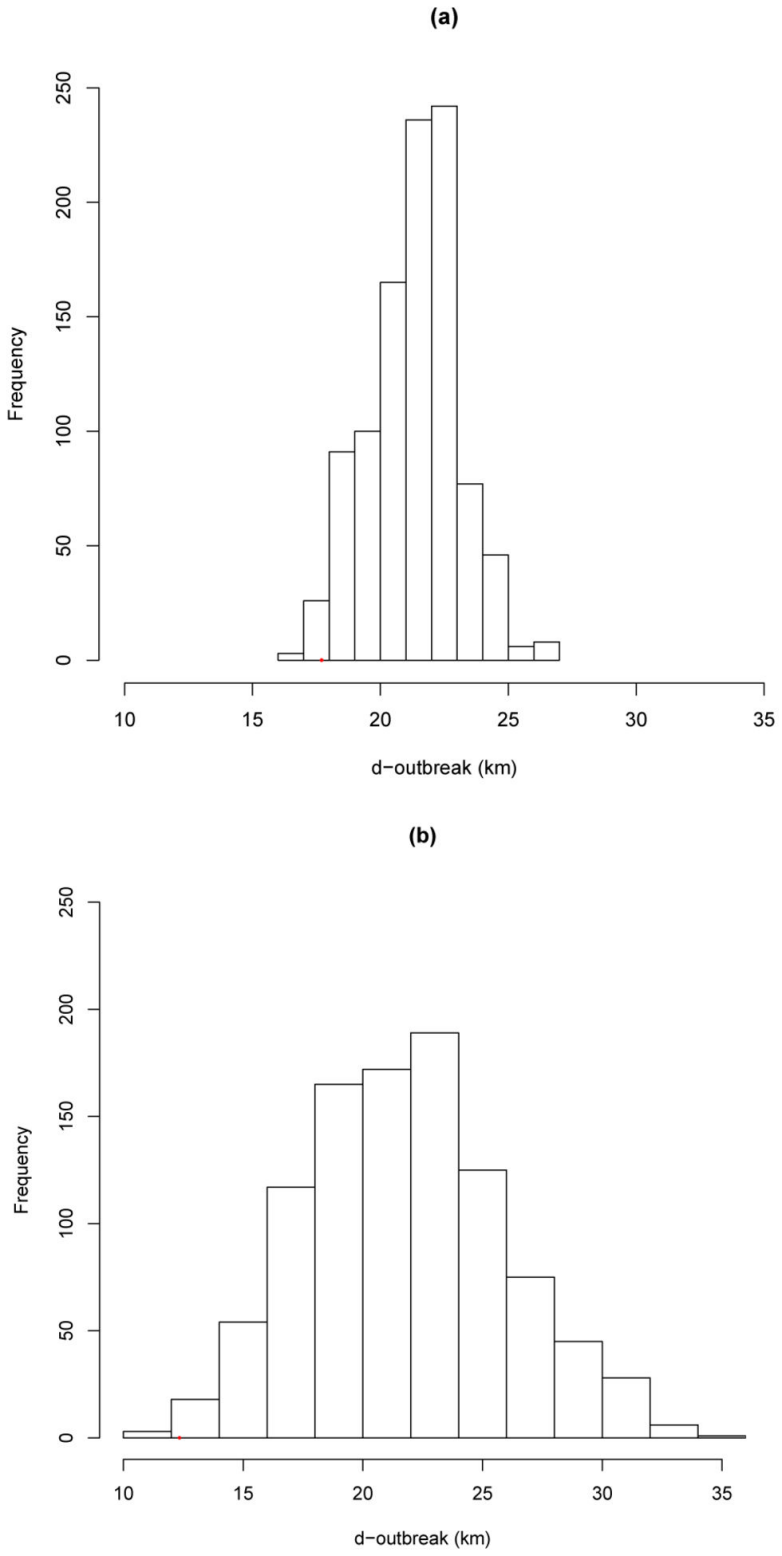
Distribution of average d-outbreak (in kilometers) obtained by bootstarp analysis for seropositve wild boar (a) using the 0.2 cut-off, and (b) using the 0.5 cut-off. Histogram shows the distribution of simulated average d-outbreak under the null hypothesis (no relationship between seropositivity and d-outbreak). Bootstrap test was for 1000 repetitions. Red dot is the observed average d-outbreak.

With the cut-off set at 0.2, 163 wild boar had an ELISA positive reaction, indicating a global apparent exposure of wild boar to MTC of 7.8% (IC_95%_: 6.7-9.1) and a true seroprevalence of 6.2% (IC_95%_: 4.6%-7.9%). Seropositive wild boar originated from 44 out of the 58 sampled “départements” (76%) (Table S1). Seven of these “départements” (16%) were cattle TB free for the same period. No significant difference was found in seropositivity between juveniles and adults (Fisher’s test, p = 0.289), or between males and females (Fisher’s test, p = 0.154). Figure 1.a shows the spatial distribution of the bPPD-ELISA positive wild boar, overlapped with the distribution of cattle outbreaks between 2000 and 2010. The d-outbreak for seropositive wild boar ranged from 0 to 103 km (mean = 24 km, median = 18 km) and from 0 to 142 km for seronegative wild boar (mean = 27 km, median = 22 km) (Figure 2.a). The average d-outbreak in positive wild boar was significantly lower than under the null hypothesis (boostrap analysis, p = 0.013) (Figure 3.a). 

With the cut-off set at 0.5, 30 wild boar exhibited an ELISA positive reaction, indicating a global apparent exposure of wild boar to MTC of 1.4% (IC_95%_: 1.0-2.1) and a true seroprevalence of 2.2% (IC_95%_: 1.5-3.2) for the whole studied area. Seropositive wild boar originated from 12 out of the 58 sampled “départements” (21%) (Table S1). Except for one department (Rhône; Figure S1), all these “départements” were those for which at least one cattle TB outbreak has been reported since 2000. No significant difference was found between seropositivity in juveniles and in adults (p = 0.076) or between males and females (p = 0.193). Figure 1.b shows the spatial distribution of the positive wild boar in bPPD-ELISA overlapped with the distribution of cattle outbreaks between 2000 and 2010. The d-outbreak for seropositive wild boar ranged from 0 to 31 km (mean = 13 km, median = 12 km) and from 0 to 142 km for seronegative wild boar (mean = 27 km, median = 22 km) (Figure 2.b). The average d-outbreak in positive wild boar was significantly lower than under the null hypothesis (boostrap analysis, p = 0.001) (Figure 3.b). 

## Discussion

The use of an ELISA to detect MTC antibodies in wild boar has permitted for the first time the description of geographic distribution of MTC contact in wild boar at a large-scale in France. Our results show that exposure of wild boar to MTC is consistent with TB outbreaks in cattle, especially when interpreting optical density with the 0.5 threshold. Moreover, using this threshold, the mean (median, respectively) distance between a seropositive wild boar and the nearest cattle outbreak is 13 km (12 km) which is compatible with the daily movement capacities of a wild boar (from 1 to 16 km, [28]) or their dispersal distances (4.90+/−5.65 km for males, reaching 38km for some individuals, in [29]; 16.6 km for males, in [30]).

The samples used for the present study were not collected for TB investigation and do not cover the whole country. In particular, some TB infected “départements” such as Dordogne (Figure S1), where several cattle outbreaks still occur and where wildlife cases are regularly discovered [8] are not included. Although our results seem to show some aggregation among seropositive wild boar, the lack of homogeneity in the sampling design of our study did not allow us to perform cluster analysis on wild boar seroprevalence data. Moreover, our results can neither be interpreted as representative of the real exposure of wild boar to MTC, nor as reflecting an accurate picture of the French situation. However, they highlight that wild boar were exposed to MTC since at least year 2000 in areas were TB is still present or has re-emerged in cattle. Most of these areas correspond to locations where TB infection has been discovered in wild individuals mainly in wild boar, red deer and/or badgers during the last 10 years [8,31]. Moreover, in these regions characterised by an important production of beef cattle, breeding is mainly extensive and farms operated over several premises with large land coverage and a long stay in pastures. This situation favors neighboring risk and increases the risk of environmental contamination mainly by interaction and contact with wildlife.

The threshold recommended by the manufacturer of the ELISA kit, 0.2, was established with Spanish data from studies on the circulation of *M. bovis* in wildlife populations. It provides a sensitivity of 72.6% and a specificity of 96.4%, and enables an early detection of TB [19]. In a recent study using this threshold in Spanish wild boar [22], seropositive animals were found in areas that were considered TB-free beforehand (North Atlantic area). As a result, surveillance was reinforced in these areas, and the first TB confirmed cases (by pathology and culture) were discovered, implying that a more intensive surveillance based on serology as a first-line diagnostic tool is likely to reveal a more widespread TB distribution [Gortazar C., personal communication). These studies suggest that in the Spanish epidemiological context, the bPPD-ELISA (with the 0.2 cut-off) can be used as an indicator of MTC contact in wild boar and as a tool to determine the areas where wildlife TB surveillance should be increased. In France, the epidemiology of MTC might be different from that of South-central Iberian Peninsula. Indeed, some seropositive results could result after exposure to *M. microti* rather than to *M. bovis*. In Spain, *M. microti* has never been reported in wild or domestic animals until present. When using the 0.2 cut-off in the present study, 162 wild boar were considered seropositive (true seroprevalence of 5.8% [4.2%-7.6%]). They originated from 76% of the sampled “départements” (44/58) among which 7 (16%) had no TB cases detected in cattle in the same period. However, wild boar from some of them showed a high seroprevalence. For instance, seroprevalence is 15.4% using the 0.2 cut-off in Côte d’Armor (Figure S1), Northern Brittany, a cattle TB free “département”. In the same communities where wild boar were sampled for our study in Côte d’Armor, several sows, an otter (*Lutra lutra*) and 2 cats were found infected by *M. microti* in the past years (M.L. Boschiroli, personal communication). MTC seropositivity in wild boar in this “département” would need to be further investigated in order to assess if our results are a first indication of *M. bovis* circulation or are a response to other MTC members. 

To obtain a higher specificity (100%), we also used a 0.5 threshold. This led to 30 seropositive wild boar, all sampled less than 31 km from a cattle outbreak, and thus, with a good consistence with TB in cattle. In particular, seropositive results with the 0.2 cut-off in wild boar from Côte d’Armor are no longer detected when the 0.5 threshold is used. However, even when using this high cut-off, we cannot ensure that the positive reaction is systematically attributed to *M. bovis* and not to other members of the MTC. Indeed, 2 of 10 piglets sampled in a farm in Côte d’Armor, where a recent outbreak of *M. microti* was discovered (detection of lesions and direct identification of the agent in two infected sows), exhibited a high serological reaction with a bPPD-ELISA index > 0.5 (EI = 0.7 and 0.8 respectively) [MLB, personal communication]. Little is known about the antibody reactivity against *M. microti* in the wild boar, although it is known to induce positive responses to TB multiantigen print immunoassay (MAPIA) and Lateral-flow-based rapid test (RT) in new World camelids [32], and positive IFN-responses to bPPD and to Lateral-flow-based rapid test (RT) in *M. microti*-infected domestic cats [33]. As previously discussed, both further investigations on *M. microti* ELISA cross-reactivity and the development of a specific test *for M. bovis* in the wild boar would be necessary. 

The results of the present study are promising and open perspectives to monitoring environmental and wild boar infection at a population level thanks to an easy, inexpensive and observer-independent tool. Indeed, given that seropositivity in wild boar was significantly related to cattle outbreaks, it can be considered as related to *M. bovis* exposure, especially when using the 0.5 cut-off. Under this condition, the ELISA test could be used as a first large-scale screening tool in TB surveillance at a population level in wild boar. 

A national surveillance program for TB in wildlife, the Sylvatub plan, has recently been launched (NOTE DE SERVICE DGAL/SDSPA/N2011-8214, Date: 20 September 2011). This program will allow us to obtain serological samples from wild boar with defined TB status in relation to the presence and severity of pathological lesions and microbiological evidence. The use of these samples will be helpful for better adapting cut-off values of the ELISA with regards to the epidemiological context of the studied region. High-risk populations, such as captive or fenced, artificially fed and/or overabundant populations, could be tested and if identified positive by ELISA they should later be surveyed in detail by combining pathology and culture.

## Supporting Information

Figure S1 **Number of wild boar tested per “département".** The numerous in each “département” indicates the number of wild boar tested by serology. “Départements” are designed by their administrative names. The red symbol locates the Brotonne Mauny forest cited in the introduction.
(TIF)

Table S1 **Apparent and true TB seroprevalence in wild boar per department and per cut-off used in ELISA bPPD.**
(XLS)
