# Supplementary figures and images for: Exposure of Wild Boar to Mycobacterium tuberculosis Complex in France since 2000 Is Consistent with the Distribution of Bovine Tuberculosis Outbreaks in Cattle
Source: PLoS One. 2013 Oct 22;8(10):e77842. doi: 10.1371/journal.pone.0077842 (PMC3805591; doi:10.1371/journal.pone.0077842)

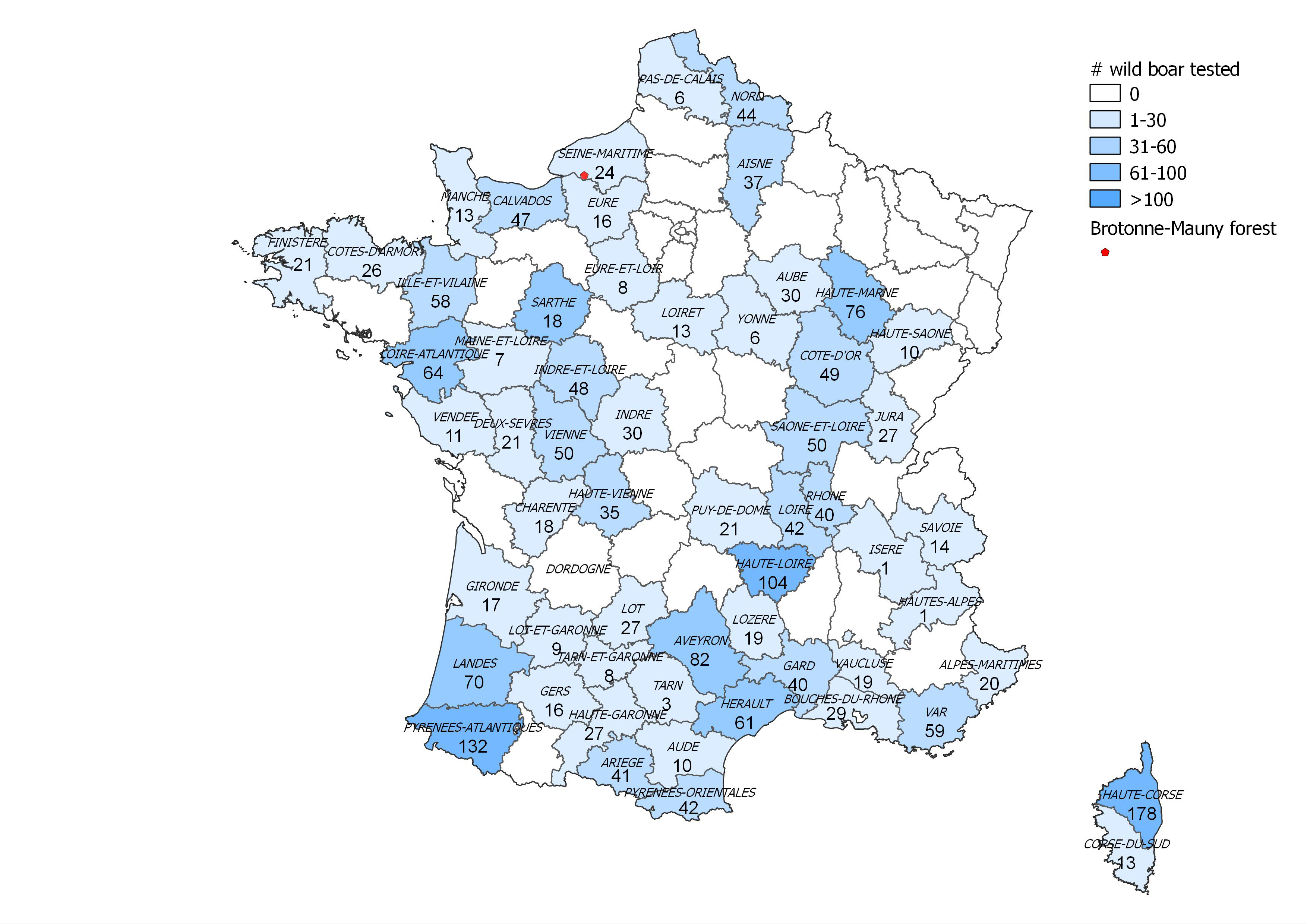

Supplement: Figure S1 — Number of wild boar tested per “département". The numerous in each “département” indicates the number of wild boar tested by serology. “Départements” are designed by their administrative names. The red symbol locates the Brotonne Mauny forest cited in the introduction. (TIF) [file pone.0077842.s001.tif]
